# Association between Dynapenic abdominal obesity and fall risk among older adults: a longitudinal study in Birjand

**DOI:** 10.1007/s40520-025-03092-7

**Published:** 2025-06-28

**Authors:** Hadiseh Rahimi Chaksari, Pouya Ebrahimi, Tahereh Yavari, Farshad Sharifi, Pedram Ramezai, Reza Pirdehghan, Fatemeh Naderi, Mitra Moodi, Masoumeh Khorashadizadeh, Moloud Payab, Mahbube Ebrahim Pour

**Affiliations:** 1 Endocrinology and Metabolism Research Center, Endocrinology and Metabolism Clinical Sciences Institute, Tehran University of Medical Sciences (TUMS), Tehran, Iran; 2 Cardiovascular Disease Research Institute, Tehran Heart Center, Tehran University of Medical Sciences, Tehran, Iran; 3 Department of Internal Medicine, Shariati Hospital, Tehran University of Medical Sciences, Tehran, Iran; 4 Elderly Health Research Center, Endocrinology and Metabolism Population Sciences Institute, Tehran University of Medical Sciences, Tehran, Iran; 5 Department of Gerontology, School of Rehabilitation, Tehran University of Medical Sciences, Tehran, Iran; 6 Faculty of Medicine, Shahid Beheshti University of Medical Sciences, Tehran, Iran; 7 Pediatric Cardiovascular Research Center, Cardiovascular Research Institute, Isfahan University of Medical Sciences, Isfahan, Iran; 8 Geriatric Health Research Center, Birjand University of Medical Sciences, Birjand, Iran; 9 Social Determinants of Health Research Center, Birjand University of Medical Sciences, Birjand, Iran; 10 Non-Communicable Diseases Research Center, Endocrinology and Metabolism Population Sciences Institute, Tehran University of Medical Sciences, Tehran, Iran

**Keywords:** Abdominal obesity, Aged, Fall, Geriatrics, Muscle strength

## Abstract

**Introduction:**

Dynapenic abdominal obesity (DAO) is the coexistence of obesity and dynapenia, defined as muscle weakness. Both abdominal obesity and dynapenia may contribute to falls in older adults. This study assesses the relationship between DAO and the risk of falls in the population ≥ over 60 who participated in the Birjand Longitudinal Aged Study (BLAS) trial.

**Methods:**

This prospective cohort study involves 1,418 elderly participants aged ≥ 60. The sample, representative of the aged population BLAS program, was selected using stratified random cluster sampling. Data on fall events — including the date, time, cause, and associated injuries — were collected through a structured researcher-designed data collection form via telephone contact with the participants or their families. Clinical examination findings and paraclinical test results per the study protocol were also available to researchers.

**Results:**

Of the 1,418 participants, 697 (51.71%) were women, and 651 (48.29%) were men, with a mean age of 69.73. The presence of DAO was significantly associated with a higher risk of falls, with a coefficient of OR = 2.65 (CI 95% 1.03–6.84, *P =* 0.044). Among the participants, 757(56.2%), 422 (31.3%), and 169 (12.5%) fell in the age groups between 60 and 69, 70–79, and ≥ 80 years, respectively. Male gender (OR: 0.45, 95%CI: 0.23–0.90, P-value: 0.23), on the other hand, was linked to a lower risk of falls, and this association was statistically significant. Furthermore, a higher risk of falls was observed among those with higher scores on the “Time to Get Up and Go” test (OR: 1.80, 95%CI: 1.11–2.92, P-value: 0.16), as well as those with depression (OR: 2.13, 95%CI: 1.30–3.49, P-value: 0.003), and anemia (OR: 1.89, 95%CI: 1.02–3.50, P-value: 0.043), with coefficients of 0.59, 0.75, and 0.63, respectively, all of which were statistically significant.

**Conclusion:**

This study’s findings suggest that DAO is a significant risk factor for falls in elderly individuals. Moreover, the male gender appears to be protected against falls. In contrast, factors such as depression, higher risk based on the Time to “Get Up and go” test, and anemia are associated with elevated risk. These factors may be crucial in understanding the relationship between Dynapenic abdominal obesity and fall risk in the elderly. They can help stratify aged adults to prevent falls more efficiently.

**Clinical trial number:**

Not applicable.

**Graphical Abstract:**

**Figure:**
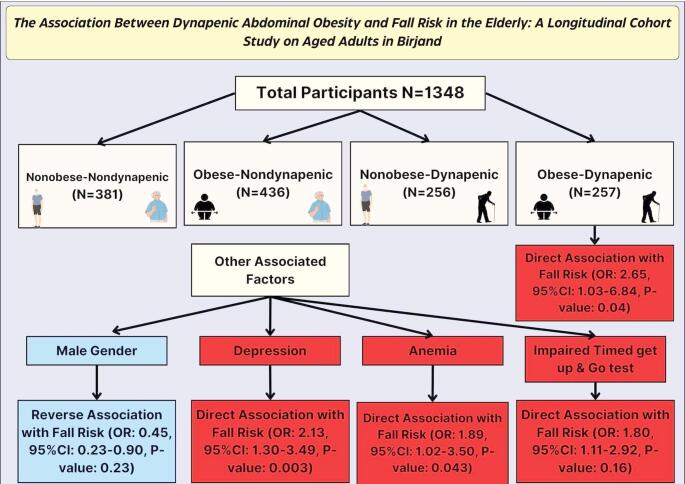
The associated factors with the risk of falls in aged adult participants

**Supplementary Information:**

The online version contains supplementary material available at 10.1007/s40520-025-03092-7.

## Introduction

Dynapenic abdominal obesity, defined as a phenotype including the cooccurrence of loss of muscle strength and abdominal obesity, is correlated with several outcomes of aging, increased morbidity, and mortality due to preventable events in previous literature [1, 2]. The crucial point in this matter is associated with several other pathological processes through fat accumulation, such as increased insulin resistance, oxidative stress, and increased risk of metabolic disorders and cardiovascular diseases [3–5]. One of the most common causes of fractures and mortality in aged populations is falling [6, 7].

The most common causes of brain injury and one of the major causes of mortality in aged adults are the falls they experience [8, 9]. Muscle weakness, lack of physical power, impairments in visual and auditory capabilities, and bone loss-induced compression fractures of the vertebrae are the major contributing factors more commonly seen in this range of ages [10]. Frailty, causing loss of muscle power and abdominal obesity, exacerbating the neurodegeneration and lack of balance, seems to be an added risk factor for these incidents [11, 12]. The relationship between muscle strength and muscle mass volume is not linear, with muscle strength diminishing faster than muscle mass [13, 14]. Evidence indicates that physical performance in older adults is more closely linked to muscle strength than muscle mass [15, 16].

Recent studies have increasingly explored the relationship between DAO and fall risk. Lv et al. (2022) [17] found that DAO was independently associated with a significantly higher risk of falls in community-dwelling older adults. Similarly, Dowling et al. [18] (2023) demonstrated that dynapenia and abdominal obesity, individually and in combination, predict increased fall risk. A prospective analysis by Veronese et al. (2023) [19] in the Osteoarthritis Initiative cohort also showed that individuals with DAO were more susceptible to future falls than those without DAO. Furthermore, Maximo et al. (2022) [6] emphasized sex-specific differences, highlighting that women with DAO may experience accelerated declines in physical performance. These findings collectively suggest that DAO is a significant and modifiable risk factor for falls, but inconsistencies across studies highlight the need for further prospective research.

The primary objective of this study was to investigate the association between DAO and the risk of falls among older adults participating in the BLAS. The secondary objectives were identifying other clinical and demographic factors associated with fall risk in this population, including depression, anemia, gait performance, and gender differences.

## Materials and methods

### Study population, sampling, and research design

This prospective cohort study was conducted on 1,418 elderly individuals ≥ over 60 who participated in the Birjand Longitudinal Adults Study (BLAS). The sampling method, a stratified random cluster sampling approach, ensures that the study results are generalizable to the elderly population of Birjand City. The study commenced in 2018, with participants enrolled from the beginning. Two rounds of telephone contact were made, with the final contact at the end of 2023. The study involved 1,418 elderly individuals aged 60 years and above, selected through stratified random cluster sampling, making the sample representative of the elderly population in Birjand. Data collection was facilitated using digital software, ensuring accuracy in sampling procedures. Participants were eligible if they were ≥ 60 and had the physical and mental capacity to participate. Excluded from the study were bedridden individuals, those with severe cognitive impairments (such as Alzheimer’s disease), or individuals with a life expectancy of less than six months. Participants were followed for a mean duration of5.2 years(range: 4.8–5.5 years) from their initial enrollment in 2018 until the final telephone contact in late 2023, during which fall events and related outcomes were recorded.

Participants were selected using stratified random cluster sampling. Strata were defined based on administrative urban districts within Birjand City to ensure geographic representativeness of the elderly population. Within each stratum, clusters were designated as primary healthcare centers, with eligible individuals randomly sampled from household registries associated with each center. This sampling framework allowed proportional representation from different geographic sectors of the region while maintaining random selection within clusters.

### Ethical considerations

Informed consent was obtained from all participants involved in the BLAS, and ethical approval was granted for this study by the research ethical committee of the Endocrinology and Metabolism Research Institute (EMRI) of Tehran University of Medical Sciences, Ref No. IR.TUMS.EMRI.REC.1396.00158). The ethical committee of Birjand University of Medical Sciences also approved the study (Ethical Code: IR. BUMS.Rec.1397.282).

### Data collection and measurements

Comprehensive questionnaires were utilized to collect demographic data such as gender, date of birth, education level, occupation, and income, along with clinical data on chronic diseases, including diabetes mellitus (DM), hypertension (HTN), and cardiovascular diseases (CVD), based on medical diagnoses.

The participants’ physical activity was assessed using the Longitudinal Ageing Study Amsterdam Physical Activity Questionnaire (LAPAQ), which evaluates physical activity during work and rest [20]. Anthropometric indices were measured according to the National Health and Nutritional Examination Survey anthropometry procedures manual Height was measured to a precision of 0.1 cm without head coverings, ensuring that the back of the head, shoulders, buttocks, and heels were aligned with the wall and that the head was in a neutral position (SECA, Germany) [21]. Weight was measured using a calibrated digital scale (SECA, Germany) with a 0.1 kg margin of error, with participants in minimal clothing. BMI was calculated as weight divided by height squared (kg/m²), and classifications followed the WHO definitions: BMI ≥ 30 as obese, 25 ≤ BMI < 30 as overweight, 18 ≤ BMI < 25 as normal, and less than 18.5 as underweight. Waist circumference was measured above the iliac crest at the navel level, with abdominal obesity defined as ≥ 102 cm for men and ≥ 88 cm for women, based on Adult Treatment Panel III (ATP III) criteria [22].

### Definition of Dynapenic abdominal obesity (DAO)

Dynapenia was defined based on handgrip strength thresholds of < 30 kg for men and < 20 kg for women, consistent with the Asian Working Group for Sarcopenia criteria and supported by prior studies evaluating dynapenia among elderly populations.

Following the Adult Treatment Panel III (ATP III) guidelines, abdominal obesity was defined as a waist circumference ≥ 102 cm for men and ≥ 88 cm for women.

These thresholds were selected to align with internationally accepted standards and to ensure comparability with previous epidemiological studies assessing fall risk in older adults. Although ethnicity-specific cut-offs may vary, the ATP III criteria are commonly applied in Middle Eastern populations, and prior Iranian studies have successfully validated their use.

Handgrip strength was measured in both hands using a digital dynamometer, with three trials for each hand. Testing began with the dominant hand, followed by a 15-second rest before the non-dominant hand was tested. Participants were categorized into four groups based on their status: (1) Individuals without abdominal obesity or dynapenia, (2) Individuals with abdominal obesity but without dynapenia, (3) Individuals without abdominal obesity but with dynapenia, and (4) Individuals with both abdominal obesity and dynapenia (DAO).

### Data collection tools

Demographic and anthropometric data and other necessary information were collected using validated questionnaires. Following the study protocol, clinical examination results and paraclinical assessments were made available to the researchers. Details regarding falls, including the date and time, causes, and resulting injuries such as fractures, hospitalizations, or minor injuries, were collected through a researcher-designed questionnaire via telephone contact with the participants or their families.

### Minimizing recall bias in data gathering

The research team implemented several strategies to minimize recall bias in telephone data collection. They designed questions with short recall periods, objective fact-based wording, and anchoring techniques to enhance memory accuracy. To reduce cognitive load, they kept to queries simple and structured, used multiple-choice responses, and conducted calls with a brief duration at optimal times. Measures to mitigate social desirability bias included ensuring anonymity, neutral wording, and cross-validating responses through alternative data sources and repeated questions. Additionally, trained interviewers avoided leading prompts and encouraged honest responses. These efforts collectively improved data accuracy and reliability. Additionally, data were cross-validated with family members or medical records when available.

### Fall risk assessment

Fall risk was evaluated using the Performance-Oriented Mobility Assessment (POMA) and the Tinetti Test. This assessment assesses both balance and gait. The POMA consists of a balance subscale (maximum 16 points) and a gait subscale (maximum 12 points), for a total maximum score of 28 points (Supplementary Tables 2 and 3).

Participants were categorized based on their total POMA scores into three fall risk levels:

- **High risk**: score < 18 points,
- **Moderate risk**: score 19–23 points,
- **Low risk**: score ≥ 24 points.

The assessment included sitting balance, rising from a chair, standing balance, turning, and evaluating step length, symmetry, and path during walking. Higher scores indicate better balance and mobility performance and a lower risk of falls [23, 24].

Handgrip strength was measured three times in each hand using a calibrated digital dynamometer. Testing began with the dominant hand, followed by a 15-second rest before testing the non-dominant hand. The highest value recorded across all six trials (three per hand) was used for the analysis. Dynapenia was defined as a maximum handgrip strength < 30 kg for men and < 20 kg for women.Gait speed was measured with a four-meter walk test, in which participants were asked to walk a distance of 4.57 m twice, with the faster walking time recorded. Slow walking speed was defined as less than 1 m per second. Blood pressure was measured twice, with a five-minute interval between readings, using an automatic blood pressure monitor. The average of the two measurements was recorded. Smoking status was evaluated using a validated questionnaire, assessing current smoking habits, smoking history, the number of cigarettes smoked per year, and exposure to secondhand smoke. Nutritional status was determined using the Mini Nutritional Assessment-Short Form (MNA-SF), with scores of 11 or less indicating a risk of malnutrition. The MNA, validated for the Iranian population, consists of 16 items divided into two sections for screening and assessing malnutrition in the elderly [25, 26]. Information regarding the consumption of common Iranian food items was collected from participants or their companions [27].

### Data analysis method

Variables included in the multivariate logistic regression models were selected based on statistically significant associations (*P* < 0.05) in univariate analyses and established clinical relevance. Age, sex, Timed Up and Go (TUG) test scores, depression status (PHQ-9), and anemia were included as adjustment factors to account for key demographic, functional, psychological, and hematological influences. Logistic regression models assessed the association between body composition groups (non-obese/non-dynapenic, obese/non-dynapenic, non-obese/dynapenic, and obese/dynapenic) and fall risk, with the non-obese/non-dynapenic group as the reference. The dependent variable was fall occurrence (yes/no) during follow-up. Variables were selected to optimize model stability and minimize overfitting.

### Handling of missing data and sensitivity analyses

Missing data were minimal (< 10%) and handled using complete case analysis. No imputation was performed. Sensitivity analyses were conducted by stratifying participants by sex (male vs. female) and age group (60–69, 70–79, ≥ 80 years) to examine whether the association between DAO and fall risk was consistent across subgroups.

## Results

**Fig. 1:**
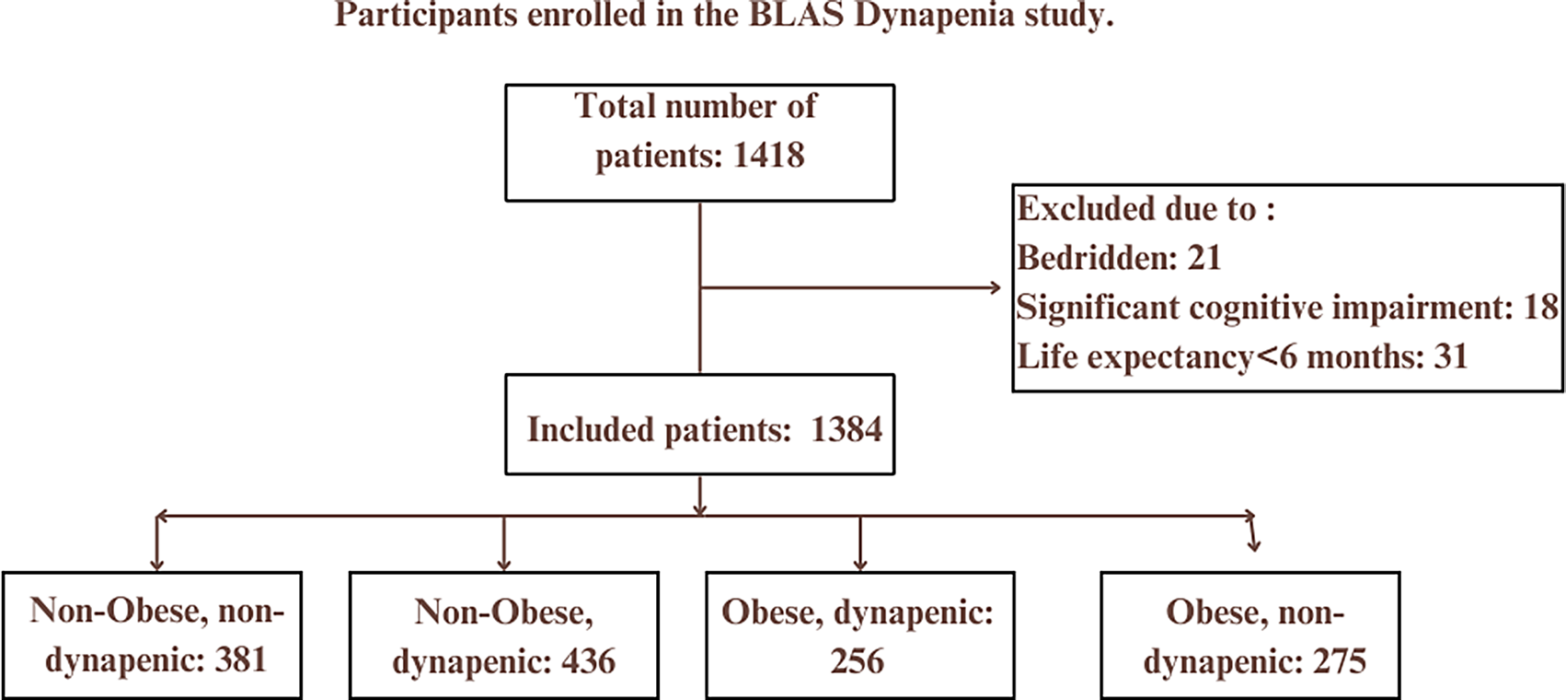
Flow chart of the study process

Out of 1,418 total planned study participants, 1348 participated, and 70 patients were excluded due to being bedridden, impaired cognitive status, or life expectancy < 6 months **(**Fig. 1**)**. Of the 1,348 participants included at baseline, 1,275 (94.6%) completed the follow-up. A total of 45 participants (3.3%) were lost to follow-up, primarily due to migration or loss of contact, and 28 participants (2.1%) died during the 6-year follow-up period. In this study, all of the respondents were living in urban areas. Among the total study participants, about 697 (51.7%) were female, and 651 (48.3%) were male, which makes the sex ratio 0.93. The mean age of the study participants was 69.73 ± 7.53 (min:60, max:96 yrs), and about 757 (56.2%), 422 (31.3%), and 169 (12.5%) of the study participants fell in the age groups between 60 and 69, 70–79 and ≥ 80 years, respectively. Out of the total study participants, 1102 (81.8%) were currently married, 606 (44.9%) were illiterate, and 515 (38.20%) and 285 (21.14%) were overweight and obese, respectively. In addition, 239 (17.7%) had smoking practices, and 161 (11.9%) were living alone. 150 (11.2%) had hearing loss, 360 (26.8%) had visual impairment, 79 (6.2%) had faller, 626 (46.65%) had gate speed less than one meter per second, and 268 (19.9%) had PHQ depressed mood. Also, 377 (27.9%) and 125 (9.3%) had mild cognitive impairment and dementia, respectively. Of the 1,348 participants included in the analysis,**79 individuals (5.9%)**experienced at least one fall during the 5-year follow-up period. Timed get up and go high risk of fall was reported in 376 (27.89%), polypharmacy 207 (15.4%), anemia 162 (12.0%), and Low serum vitamin D 716 (56.0%). Risk of malnutrition and malnutrition were reported in 344 (25.5%) and 15 (1.1%), respectively ***(***Table 1***).***

**Table 1:** Prevalence of obesity and dynapenia of study participants

Category	Total (*n* = 1348)	Non-obese, non-dynapenic (*n* = 381)	Obese, non-dynapenic (*n* = 436)	Non-obese, Dynapenic (*n* = 256)	Obese, Dynapenic (*n* = 275)
**Demographics**					
Age, mean ± SD (years)	69.7 ± 7.5	-	-	-	-
Female, n (%)	697 (51.7%)	66 (17.3%)	329 (75.5%)	67 (26.2%)	235 (85.5%)
Married, n (%)	1102 (81.8%)	352 (92.4%)	357 (81.9%)	200 (78.4%)	—
Living alone, n (%)	161 (11.9%)	20 (5.3%)	64 (14.7%)	23 (9.0%)	—
**Anthropometric Measures**					
Gait speed < 1 m/s, n (%)	626 (46.7%)	74 (19.5%)	226 (52.0%)	139 (54.7%)	187 (68.5%)
**Clinical Variables**					
Depression (PHQ-9), n (%)	268 (19.9%)	51 (13.5%)	84 (19.3%)	60 (23.4%)	73 (26.6%)
Anemia, n (%)	162 (12.0%)	44 (11.6%)	35 (8.0%)	52 (20.3%)	31 (11.3%)
Dementia (6-CIT), n (%)	125 (9.3%)	24 (6.3%)	20 (4.6%)	46 (18.0%)	35 (12.7%)
Visual impairment, n (%)	360 (26.9%)	98 (25.8%)	112 (25.9%)	71 (28.0%)	79 (28.7%)
Hearing loss, n (%)	150 (11.2%)	29 (7.6%)	35 (8.1%)	59 (23.0%)	27 (9.8%)
**Functional Status**					
High fall risk (TUG test < 18), n (%)	376 (27.9%)	69 (18.1%)	114 (26.2%)	94 (36.7%)	99 (36.0%)
**Lifestyle Factors**					
Current/ex-smoker, n (%)	239 (17.7%)	104 (27.3%)	42 (9.6%)	69 (27.0%)	24 (8.7%)
Physical inactivity, n (%)	661 (49.1%)	140 (36.8%)	219 (50.2%)	135 (52.7%)	167 (60.7%)

In the unadjusted model, compared to individuals without obesity and dynapenia, the presence of either condition alone or combined was associated with an increased risk of falls. Specifically, obese non-dynapenic individuals had an odds ratio (OR) of 4.10 (standard error [SE] 1.74; 95% confidence interval [CI] 1.79–9.42; *P* = 0.001), while non-obese dynapenic individuals had an OR of 3.44 (SE 1.60; 95% CI 1.38–8.59; *P* = 0.008). The association was strongest among individuals with both obesity and dynapenia (obese dynapenic group), who had an OR of 5.42 (SE 2.36; 95% CI 2.30–12.74; *P* < 0.001).

After adjusting for potential confounders, including age, sex, Timed Up and Go (TUG) test score, depressed mood, and anemia, the strength of these associations attenuated. In the adjusted model, obese non-dynapenic individuals had an OR of 2.42 (SE 1.12; 95% CI 0.97–6.01; *P* = 0.057), and non-obese dynapenic individuals had an OR of 2.45 (SE 1.16; 95% CI 0.96–6.22; *P* = 0.060), both approaching but not reaching conventional statistical significance. However, the association remained statistically significant for individuals with both obesity and dynapenia, with an adjusted OR of 2.65 (SE 1.28; 95% CI 1.03–6.84; *P* = 0.044).

Regarding covariates, male sex was a protective factor against falls (OR 0.45; SE 0.16; 95% CI 0.23–0.90; *P* = 0.023). Higher TUG test times, indicating poorer mobility, were associated with an increased fall risk (OR 1.80; SE 0.44; 95% CI 1.11–2.92; *P* = 0.016). Additionally, the presence of depressed mood (OR 2.13; SE 0.54; 95% CI 1.30–3.49; *P* = 0.003) and anemia (OR 1.89; SE 0.59; 95% CI 1.02–3.50; *P* = 0.043) were also significantly associated with a higher risk of falls (Tables 2 and 3)*.*

In sensitivity analyses stratified by sex and age group, the association between DAO and increased fall risk remained consistent. In both male and female participants and across all age categories, DAO was associated with a higher risk of falls, although effect sizes varied slightly (Supplementary Table 3).

**Table 2:** Uni and multivariate association of obesity and dynapenia with fall

	OR (SE)	95% CI OR	*P* value
**Unadjusted model**			
Non obese non dynapenic			
obese nondynapenic	4.10 (1.74)	1.79–9.42	0.001
nonobese dynapenic	3.44 (1.60)	1.38–8.59	0.008
obese dynapenic	5.42 (2.36)	2.30-12.74	0.000
**Adjusted model**			
Nonobese non dynapenic			
Obese nondynapenic	2.42 (1.12)	0.97–6.01	0.057
Nonobese dynapenic	2.45 (1.16)	0.96–6.22	0.060
Obese dynapenic	2.65 (1.28)	1.03–6.84	0.044
Sex	0.45 (0.16)	0.23–0.90	0.023
TUG	1.80 (0.44)	1.11–2.92	0.016
Depressed mood	2.13 (0.54)	1.30–3.49	0.003
Anemia	1.89 (0.59)	1.02–3.50	0.043

**Table 3:** Time to event Uni and multivariate association of obesity and dynapenia with fall

	HR (SE)	95% CI OR	*P* value
**Unadjusted model**			
Nonobese non dynapenic			
Obese nondynapenic	3.77 (1.58)	1.65–8.58	0.002
Nonobese dynapenic	3.29 (1.50)	1.34–8.09	0.009
Obese dynapenic	5.24 (2.24)	2.26–12.10	0.000
**Adjusted model 1**			
Nonobese nondynapenic			
Obese nondynapenic	2.07 (0.94)	0.97–6.01	0.109
Nonobese dynapenic	2.53 (1.21)	0.96–6.22	0.051
Obese dynapenic	2.56 (1.20)	1.03–6.84	0.045
Age	1.02 (0.01)	0.98–1.05	0.196
Sex	0.35 (0.11)	0.19–0.67	0.001
**Adjusted model 2**			
Nonobese non dynapenic			
Obese nondynapenic	2.23 (0.94)	0.91–5.45	0.079
Nonobese dynapenic	2.33 (1.21)	0.94–5.79	0.069
Obese dynapenic	2.51 (1.17)	1.00-6.29	0.049
Sex	0.45 (0.15)	0.24–0.87	0.017
TUG	1.66 (0.39)	1.04–2.64	0.033
Depressed mood	2.02 (0.48)	1.27–3.23	0.003
Anemia	1.49 (0.45)	0.83–2.69	0.179

## Discussion

The present study categorizes the research population into four distinct groups based on the presence or absence of abdominal obesity and dynapenia. The analysis highlights the differences among these groups regarding demographic variables, physical and psychological conditions, and laboratory findings, focusing primarily on identifying factors that increase the risk of falls within this population.

It was observed that individuals with dynapenia tend to be older than their non-dynapenic counterparts, which aligns with expectations since aging is a primary contributor to muscle loss. This underscores the need for heightened clinical attention to this subgroup, particularly those with concurrent abdominal obesity [6, 28]. Our findings suggest that individuals with both abdominal obesity and dynapenia are at a significantly higher risk of falls.

Several plausible biological and functional mechanisms may explain the observed association between DAO and increased fall risk among older adults. Dynapenia, characterized by reduced muscle strength, compromises an individual’s balance control, reaction time, and ability to recover from perturbations, all critical to preventing falls [29]. Meanwhile, abdominal obesity alters body biomechanics by anteriorly shifting the center of gravity, leading to postural instability and impaired gait [30]. The coexistence of dynapenia and abdominal obesity may synergistically worsen neuromuscular control, increasing the load on weakened lower limb muscles and impairing proprioceptive feedback [6, 19]. Furthermore, abdominal obesity is associated with systemic inflammation, oxidative stress, and insulin resistance, which can accelerate sarcopenia progression, exacerbate muscle weakness, and impair neural pathways involved in motor control [4, 31]. Together, these mechanisms likely contribute to the increased susceptibility to falls observed in individuals with DAO. Understanding these interconnected pathways emphasizes the need for comprehensive interventions that simultaneously target muscle strengthening, balance retraining, and abdominal fat reduction to mitigate fall risk.

While age is a non-modifiable factor in the association between abdominal obesity, dynapenia, and fall risk, it remains a critical risk factor. Older adults, particularly those suffering from both abdominal obesity and dynapenia, should be carefully monitored to mitigate the risk of falls [17, 32]. Furthermore, body mass index (BMI) and waist circumference were notably higher among individuals with abdominal obesity, as expected. However, the regression analysis revealed that neither abdominal obesity nor dynapenia significantly increased fall risk. It is the coexistence of both conditions—abdominal obesity and dynapenia—that substantially elevates the likelihood of falls, highlighting the need for focused clinical interventions for this group [19].

Muscle strength and walking speed were higher in individuals without dynapenia, which directly reflects the detrimental impact of dynapenia on these functional parameters. The increased fall risk in elderly individuals with abdominal obesity and dynapenia may be attributed to the impairment of muscle strength and walking speed [6, 33]. Consequently, interventions targeting any of these factors could have a beneficial cascading effect on related factors, offering a more holistic approach to fall prevention [33, 34].

Our findings also show a higher prevalence of abdominal obesity with dynapenia among women, particularly those aged over 80, underscoring the greater vulnerability of older women to dynapenia-related complications. These individuals require tailored interventions to reduce the fall risk and its associated consequences [35, 36]. Women exhibit a higher prevalence of obesity, further exacerbating their fall risk, as demonstrated by the regression analysis [35]. Interestingly, gender was found to act as a protective factor in this model, with men showing a lower likelihood of falls, particularly in elderly populations [35, 36].

Illiteracy was more prevalent in individuals with both abdominal obesity and dynapenia, pointing to the need for educational interventions tailored to their cognitive and social capacities. Such interventions, particularly those delivered through caregivers or community-based programs, could raise awareness and promote preventive measures [37]. Additionally, a higher proportion of individuals with abdominal obesity in the dynapenia group were either single or living alone, suggesting insufficient social support. This finding emphasizes the need for enhanced community care programs to actively monitor and assist these vulnerable individuals [38, 39].

Interestingly, the prevalence of smoking was lower in individuals with abdominal obesity, potentially due to smoking-related appetite suppression, which may reduce obesity rates in these individuals [40]. However, other resources show the reverse outcomes and conclude that smoking has been associated with increased abdominal circumference and waist circumference [41]. Visual and hearing impairments, as well as a history of falls, were more frequently observed in individuals with abdominal obesity and dynapenia, likely due to their advanced age and physical impairments. These sensory deficits further elevate their fall risk, necessitating thorough physical evaluations and timely interventions [42–44].

Psychologically, depression and dementia were more prevalent among individuals with dynapenia, indicating the importance of addressing mental health alongside physical care to reduce fall risk in this group [45, 46]. Dynapenia was also associated with higher malnutrition rates, anemia, and sedentary behavior, which compound fall risk [47, 48]. The presence of depression and anemia was significantly correlated with increased fall risk, as measured by the “Timed Up and Go” test, highlighting the need for psychological and nutritional interventions. However, Barry mentions several studies. E. et al., in a systematic review, showed this test as an unaccountable predictor of falls [49].

While our findings align with several previous studies demonstrating an increased fall risk associated with DAO, some studies have reported inconsistent or null associations. For example, Dowling et al. (2023) [18] found that while dynapenia alone was a strong predictor of falls, abdominal obesity did not independently predict fall risk when muscle strength was preserved. Similarly, Maximo et al. (2022) [50] reported that the association between DAO and physical decline varied by sex, with a stronger effect observed in women than men, suggesting potential heterogeneity. Differences in study populations, including baseline physical activity levels, nutritional status, and comorbidities, may explain these discrepancies. Moreover, methodological differences such as the definition of dynapenia (e.g., absolute vs. relative strength thresholds), the criteria for obesity (e.g., BMI vs. waist circumference), and fall assessment methods (self-reported falls vs. medically documented falls) could contribute to the observed inconsistencies across studies. These findings underscore the need for standardized diagnostic criteria and longitudinal studies across diverse populations better to understand the complex relationship between DAO and fall risk.

In the regression analysis conducted in this study, male gender was identified as a protective factor against falls. In contrast, modifiable risk factors such as depression and anemia were significant predictors of falls. Interventions targeting these modifiable factors could reduce fall risk among individuals with abdominal obesity and dynapenia. However, further interventional studies are necessary to assess the effectiveness of such interventions.

## Conclusion

The findings of this study indicate a significant association between abdominal obesity and dynapenia and fall risk in elderly individuals. The male gender acts as a protective factor, while modifiable risk factors like depression and anemia increase fall risk. Given the increased risk of falls among individuals with both conditions, targeted interventions focused on reducing abdominal obesity, enhancing muscle strength, and addressing psychological and nutritional health are crucial. Further studies are needed to explore the efficacy of such interventions in fall prevention.

### Study limitations

This study has several limitations that should be acknowledged. First, the cohort design limits the ability to establish a strong causal relationship between DAO and fall risk, despite the prospective data collection. Future prospective and interventional studies are recommended to validate these findings and explore causality.

Second, fall data were collected via telephone interviews, introducing a potential for recall bias. Although we employed multiple strategies to minimize bias, including using short recall periods, structured questionnaires, and trained interviewers, some degree of underreporting or misclassification may persist.

Third, despite adjusting for several confounders, residual confounding from unmeasured variables such as medication use (e.g., sedatives), frailty status, or sensory impairments may still affect the observed associations.

Fourth, the study population consisted entirely of older adults from a single geographic region in Iran (Birjand), which may limit the generalizability of the findings to other populations with different ethnic, cultural, or environmental characteristics.

## Electronic supplementary material

Below is the link to the electronic supplementary material.

Supplementary Material 1

## Data Availability

Further data will be provided upon reasonable request by the corresponding author.

## Abbreviations

DAO: Dynapenic abdominal obesity
BLAS: Birjand Longitudinal Aged Study
UN: United Nations
WHO: World Health Organization
EMRI: Endocrinology and Metabolism Research Institute
LAPAQ: Longitudinal Ageing Study Amsterdam Physical Activity Questionnaire
DM: Diabetes Mellitus
HTN: Hypertension
CVD: Cardiovascular diseases
BMI: Body Mass Index
NHANES: National Health and Nutritional Examination Survey
MNA-SF: Mini Nutritional Assessment- Short Form
PHQ-9: Patient Health Questionaire-9
POMA: Performance-Oriented Mobility Assessment
6-CIT: Six-Item Cognitive Impairment Test

## References

[1] Zhou S, Luo N, Si H, Da W, Liu Y, Wu L et al (2024) Association between dynapenic abdominal obesity and arthritis among the middle-aged and older chinese: a longitudinal study. Aging Clin Exp Res 36(1):198 39367987 10.1007/s40520-024-02847-y PMC11455664

[2] Rahimi Farahani M, Sharifi F, Payab M, Shadman Z, Fakhrzadeh H, Moodi M et al (2024) Dynapenia-abdominal obesity and mortality risk, is independent effect obscured by age and frailty?Birjand longitudinal aging study (BLAS). J Diabetes Metab Disord 23(2):2343–2353 39610561 10.1007/s40200-024-01501-8 PMC11599648

[3] Axelrod CL, Dantas WS, Kirwan JP (2023) Sarcopenic obesity: emerging mechanisms and therapeutic potential. Metabolism 146:155639 37380015 10.1016/j.metabol.2023.155639 PMC11448314

[4] Hong Shyeon, Choi KM (2020) Sarcopenic obesity, insulin resistance, and their implications in cardiovascular and metabolic consequences. Int J Mol Sci 21(2):494 31941015 10.3390/ijms21020494 PMC7013734

[5] Wang X, Jiang J, Hu W, Hu Y, Qin LQ, Hao Y et al (2023) Dynapenic abdominal obesity and risk of heart disease among Middle-Aged and older adults: A prospective cohort study. J Nutr Health Aging 27(9):752–75837754215 10.1007/s12603-023-1975-0

[6] Zhang L, Liu S, Wang W, Sun M, Tian H, Wei L et al (2022) Dynapenic abdominal obesity and the effect on long-term gait speed and falls in older adults. Clin Nutr 41(1):91–96 34864458 10.1016/j.clnu.2021.11.011

[7] Attar M, Alsinnari YM, Alqarni MS, Bukhari ZM, Alzahrani A, Abukhodair AW et al (2021) Common Types of Falls in the Elderly Population, Their Associated Risk Factors and Prevention in a Tertiary Care Center. Cureus 10.7759/cureus.14863 PMC8184103 34113501

[8] Toutounji Z, Alahmad Z, Attar M, Sarminy M, Alsado WM, Mohammad M (2024) Unusual location of Gastrointestinal stromal tumor (GIST): A case report and literature review of greater omentum location. Int J Surg Case Rep 119:109793 38781841 10.1016/j.ijscr.2024.109793 PMC11143788

[9] Moreland BL, Kakara R, Haddad YK, Shakya I, Bergen G (2021) A descriptive analysis of location of older adult falls that resulted in emergency department visits in the united states, 2015. Am J Lifestyle Med 15(6):590–597 34916877 10.1177/1559827620942187 PMC8669898

[10] Appeadu MKBB Falls and Fall Prevention in Older Adults. [Updated 2023 Jun 4]. In: StatPearls [Internet]. Treasure Island (FL): StatPearls Publishing; 2024. Available from: https://www.ncbi.nlm.nih.gov/books/NBK560761 32809596

[11] Yang ZC, Lin H, Jiang GH, Chu YH, Gao JH, Tong ZJ et al (2023) Frailty is a risk factor for falls in the older adults: A systematic review and Meta-Analysis. J Nutr Health Aging 27(6):487–49537357334 10.1007/s12603-023-1935-8

[12] Mazon JN, de Mello AH, Ferreira GK, Rezin GT (2017) The impact of obesity on neurodegenerative diseases. Life Sci 182:22–28 28583368 10.1016/j.lfs.2017.06.002

[13] Goodpaster BH, Park SW, Harris TB, Kritchevsky SB, Nevitt M, Schwartz AV et al (2006) The loss of skeletal muscle strength, mass, and quality in older adults: the health, aging and body composition study. J Gerontol Biol Sci Med Sci 61(10):1059–1064 10.1093/gerona/61.10.1059 17077199

[14] Cruz-Jentoft AJ, Sayer AA, Sarcopenia (2019) Lancet 393(10191):2636–2646 31171417 10.1016/S0140-6736(19)31138-9

[15] Pisciottano MVC, Pinto SS, Szejnfeld VL, de Castro CH (2014) The relationship between lean mass, muscle strength and physical ability in independent healthy elderly women from the community. J Nutr Health Aging 18(5):554–55824886744 10.1007/s12603-013-0414-z

[16] Schaap LA, Koster A, Visser M, Adiposity (2013) Muscle mass, and muscle strength in relation to functional decline in older persons. Epidemiol Rev 35(1):51–65 23221972 10.1093/epirev/mxs006

[17] Lv D, Shen S, Chen X (2022) Association between dynapenic abdominal obesity and fall risk in older adults. Clin Interv Aging 17:439–445 10.2147/CIA.S347053 PMC9001023 35418747

[18] Dowling L, McCloskey E, Cuthbertson DJ, Walsh JS (2023) Dynapenic abdominal obesity as a risk factor for falls. J Frailty Aging 12(1):37–42 36629082 10.14283/jfa.2022.18

[19] Veronese N, Koyanagi A, Soysal P, Bolzetta F, Dominguez LJ, Barbagallo M et al (2023) Dynapenic abdominal obesity and susceptibility to fall: a prospective analysis of the osteoarthritis initiative. Front Nutr. 10 10.3389/fnut.2023.1153399 PMC10198130 37215209

[20] Siebeling L, Wiebers (2012) Beem, puhan, Ter riet. Validity and reproducibility of a physical activity questionnaire for older adults: questionnaire versus accelerometer for assessing physical activity in older adults. Clin Epidemiol. 171 10.2147/CLEP.S30848 PMC3410686 22866018

[21] National Health and Nutrition Examination Survey 2015 – 2018: Sample Design and Estimation Procedures 33663649

[22] Lipsy RJ (2003) The National cholesterol education program adult treatment panel III guidelines. J Managed Care Pharm 9(1 Supp A):2–5 10.18553/jmcp.2003.9.s1.2 PMC10437161 14613351

[23] Núñez-Cortés R, Cruz B, del Gallardo-Gómez P, Calatayud D, Cruz-Montecinos J, López-Gil C (2022) Handgrip strength measurement protocols for all-cause and cause-specific mortality outcomes in more than 3 million participants: A systematic review and meta-regression analysis. Clin Nutr 41(11):2473–2489 36215867 10.1016/j.clnu.2022.09.006

[24] Yang C, Mo Y, Cao X, Zhu S, Wang X, Wang X (2023) Reliability and validity of the Tinetti performance oriented mobility assessment in Chinese community-dwelling older adults. Geriatr Nurs (Minneap) 53:85–89 10.1016/j.gerinurse.2023.06.020 37454423

[25] Guigoz Y, Vellas B, Garry PJ (1996) Assessing the nutritional status of the elderly: the Mini nutritional assessment as part of the geriatric evaluation. Nutr Rev 54(1 Pt 2):S59–65 8919685 10.1111/j.1753-4887.1996.tb03793.x

[26] Kaiser MJ, Bauer JM, Ramsch C, Uter W, Guigoz Y, Cederholm T et al (2009) Validation of the Mini nutritional assessment short-form (MNA^®^-SF): A practical tool for identification of nutritional status. J Nutr Health Aging 13(9):782–78819812868 10.1007/s12603-009-0214-7

[27] Amirkalali B, Sharifi F, Fakhrzadeh H, Mirarefin M, Ghaderpanahi M, Larijani B (2010) Evaluation of the Mini Nutritional Assessment in the elderly, Tehran, Iran. Public Health Nutr. 20100301st ed.;13(9):1373–9 10.1017/S1368980010000303 20188008

[28] Choi YA, Lee JS, Kim YH (2022) Association between physical activity and dynapenia in older adults with COPD: a nationwide survey. Sci Rep 12(1):7480 35523837 10.1038/s41598-022-11504-1 PMC9076677

[29] Manini TM, Clark BC (2012) Dynapenia and aging: an update. Journals Gerontology: Ser A 67A(1):28–40 10.1093/gerona/glr010 PMC3260480 21444359

[30] Garcia PA, de Queiroz LL, Caetano MBD, Silva KHCV (2021) e, Hamu TCD da S. Obesity is associated with postural balance on unstable surfaces but not with fear of falling in older adults. Braz J Phys Ther. 25(3):311–8 10.1016/j.bjpt.2020.08.003 PMC8134782 32830064

[31] Alexandre TdaS, Scholes S, Santos JLF, de Oliveira C (2019) Dynapenic abdominal obesity as a risk factor for worse trajectories of ADL disability among older adults: the ELSA cohort study. Journals Gerontology: Ser A 74(7):1112–1118 10.1093/gerona/gly182 PMC6580691 30165562

[32] Kao CY, Su YC, Chang SF (2023) The relationship between dynapenic abdominal obesity and fall: A systematic review and Meta-Analysis of 15,506 middle to older adults. J Clin Med 12(23):7253 38068305 10.3390/jcm12237253 PMC10706955

[33] Skelton DA, Rutherford OM, Dinan-Young S, Sandlund M (2019) Effects of a falls exercise intervention on strength, power, functional ability and bone in older frequent fallers: fame (Falls management Exercise) RCT secondary analysis. J Frailty Sarcopenia Falls 04(01):11–19 10.22540/JFSF-04-011 PMC7155371 32300711

[34] Zanker J, Scott D, Alajlouni D, Kirk B, Bird S, DeBruin D et al (2023) Mortality, falls and slow walking speed are predicted by different muscle strength and physical performance measures in women and men. Arch Gerontol Geriatr 114:105084 37290229 10.1016/j.archger.2023.105084

[35] Gale CR, Cooper C, Aihie Sayer A (2016) Prevalence and risk factors for falls in older men and women: the english longitudinal study of ageing. Age Ageing 45(6):789–794 27496938 10.1093/ageing/afw129 PMC5105823

[36] Pereira CB, Kanashiro AMK (2022) Falls in older adults: a practical approach. Arq Neuropsiquiatr 80(5 suppl 1):313–323 35976297 10.1590/0004-282X-ANP-2022-S107 PMC9491436

[37] Kuhirunyaratn P, Prasomrak P, Jindawong B (2019) Effects of a health education program on fall risk prevention among the urban elderly: A Quasi-Experimental study. Iran J Public Health 48(1):103–111 30847317 PMC6401575

[38] Burton E, Lewin G, O’Connell H, Hill K (2018) Falls prevention in community care: 10 years on. Clin Interv Aging 13:261–269 29483772 10.2147/CIA.S153687 PMC5813950

[39] Pillay J, Riva JJ, Tessier LA, Colquhoun H, Lang E, Moore AE et al (2021) Fall prevention interventions for older community-dwelling adults: systematic reviews on benefits, harms, and patient values and preferences. Syst Rev 10(1):18 33422103 10.1186/s13643-020-01572-7 PMC7797084

[40] Graff-Iversen S, Hewitt S, Forsén L, Grøtvedt L, Ariansen I (2019) Associations of tobacco smoking with body mass distribution; a population-based study of 65,875 men and women in midlife. BMC Public Health 19(1):1439 31675936 10.1186/s12889-019-7807-9 PMC6825363

[41] Shi L, An R, Van Meijgaard J (2013) Cigarette smoking and abdominal obesity: a meta-analysis of observational studies. J Subst Use 18(6):440–449

[42] Gopinath B, McMahon CM, Burlutsky G, Mitchell P (2016) Hearing and vision impairment and the 5-year incidence of falls in older adults. Age Ageing 45(3):409–414 26946051 10.1093/ageing/afw022

[43] Mehta J, Czanner G, Harding S, Newsham D, Robinson J (2022) Visual risk factors for falls in older adults: a case-control study. BMC Geriatr 22(1):134 35177024 10.1186/s12877-022-02784-3 PMC8855581

[44] Wapp C, Mittaz Hager AG, Hilfiker R, Zysset P (2022) History of falls and fear of falling are predictive of future falls: outcome of a fall rate model applied to the Swiss CHEF trial cohort. Front Aging. 3 10.3389/fragi.2022.1056779 PMC9795055 36589140

[45] Teixeira IA, Coutinho ESF, Marinho V, Castro-Costa E, Deslandes AC (2023) Prevalence of dynapenia and overlap with disability, depression, and executive dysfunction. Rev Saude Publica 57(1):43 37556665 10.11606/s1518-8787.2023057004580 PMC10355316

[46] Qian S, Huang T, Wen Q, Zhang Y, Chen J, Feng X (2024) Dynapenic abdominal obesity and the risk of depressive symptoms in middle-aged and older Chinese adults: evidence from a National cohort study. J Affect Disord 355:66–72 38548204 10.1016/j.jad.2024.03.115

[47] de Blasio F, Scalfi L, Castellucci B, Sacco AM, Berlingieri GM, Capitelli L et al (2022) Poor nutritional status and dynapenia are highly prevalent in Post-Acute COVID-19. Front Nutr. 9 10.3389/fnut.2022.888485 PMC9205211 35719154

[48] Karismaz A, Pasin O, Kara O, Eren R, Smith L, Doventas A et al (2024) Associations between anemia and dependence on basic and instrumental activities of daily living in older women. BMC Geriatr 24(1):741 39244584 10.1186/s12877-024-05342-1 PMC11380193

[49] Barry E, Galvin R, Keogh C, Horgan F, Fahey T (2014) Is the timed up and go test a useful predictor of risk of falls in community dwelling older adults: a systematic review and meta- analysis. BMC Geriatr 14(1):14 24484314 10.1186/1471-2318-14-14 PMC3924230

[50] Máximo R, de O DC, Ramirez PC, Luiz MM, de Souza AF, Delinocente MLB et al (2022) Combination of dynapenia and abdominal obesity affects long-term physical performance trajectories in older adults: sex differences. Am J Clin Nutr 115(5):1290–1299 35102379 10.1093/ajcn/nqac023 PMC9071386

