# Supplementary material for: Association between Dynapenic abdominal obesity and fall risk among older adults: a longitudinal study in Birjand
Source: Aging Clin Exp Res. 2025 Jun 28;37(1):201. doi: 10.1007/s40520-025-03092-7 (PMC12206166; doi:10.1007/s40520-025-03092-7)
Supplement: Supplementary file 1 — Supplementary Material 1 [file 40520_2025_3092_MOESM1_ESM.docx]

## Supplementary Table 1. Balance Assessment (POMA-B) - 16 Points Total

| Task | Scoring |
| --- | --- |
| Sitting Balance | 0 - Unsteady 1 - Steady |
| Arising from Chair | 0 - Unable without assistance 1 - Uses arms to rise 2 - No use of arms |
| Attempts to Rise | 0 - Unable without help 1 - Requires multiple attempts 2 - Rises in one attempt |
| Immediate Standing Balance (first 5 sec) | 0 - Unsteady (staggering, grabbing support) 1 - Steady with support 2 - Steady without support |
| Standing Balance | 0 - Unsteady 1 - Steady with support 2 - Steady without support |
| Nudged Balance (light push on sternum, feet together) | 0 - Begins to fall 1 - Staggers but recovers 2 - Steady |
| Eyes Closed Balance | 0 - Unsteady 1 - Steady |
| Turning 360 Degrees | 0 - Discontinuous, unsteady steps 1 - Steady, but slow 2 - Smooth turn |
| Sitting Down | 0 - Falls into chair 1 - Uses arms for control 2 - Controlled descent |

## Supplementary Table 2. Gait Assessment (POMA-G) - 12 Points Total

| Task | Scoring |
| --- | --- |
| Gait Initiation | 0 - Hesitation 1 - No hesitation |
| Step Length & Height | 0 - Unequal steps or does not clear floor 1 - Unequal steps but clears floor 2 - Equal steps, foot clears floor |
| Step Symmetry | 0 - Unequal steps 1 - Equal steps |
| Step Continuity | 0 - Stopping or shuffling 1 - Smooth, continuous steps |
| Path Deviation | 0 - Marked deviation 1 - Mild deviation 2 - Straight path |
| Trunk Stability While Walking | 0 - Marked sway or use of aid 1 - No sway but flexes knees or widens base 2 - Stable trunk, no compensation |
| Walking Stance (Base of Support) | 0 - Heels apart 1 - Heels almost touching |

| **Subgroup** | **Odds Ratio (OR)** | **95% Confidence Interval** | **P-value** |
| --- | --- | --- | --- |
| Male | 2.10 | 1.05–4.21 | 0.035 |
| Female | 2.90 | 1.45–5.81 | 0.012 |
| Age 60–69 | 2.50 | 1.20–5.22 | 0.024 |
| Age 70–79 | 2.70 | 1.30–5.60 | 0.020 |
| Age ≥80 | 3.00 | 1.10–8.21 | 0.018 |

**Supplementary Table 3.** Association between DAO and Fall Risk Stratified by Sex and Age Group
